# Antioxidant potential of nanomaterials

**DOI:** 10.55730/1300-0152.2658

**Published:** 2023-08-10

**Authors:** David GONZÁLEZ-FLORES, Javier ESPINO, José Antonio PARIENTE

**Affiliations:** 1 Department of Anatomy, Cell Biology and Zoology, Faculty of Sciences, University of Extremadura, Badajoz, Spain; 2 Department of Physiology, Faculty of Sciences, University of Extremadura, Badajoz, Spain

**Keywords:** Reactive oxygen species, nanomaterial, oxidative stress, antioxidant

## Abstract

**Background/aim:**

The novel field of nanomaterials allows infinite possibilities in order to create antioxidant therapies. The present review is aimed to describe the state of art concerning on nanomaterials and their effects on reactive oxygen species (ROS) production. A wide range of nanoparticles has been designed for this purpose, and each one possesses some particular characteristics which allow these significant antioxidant results. Several in vivo and in vitro works state the ability of these nanoparticles to mimic the redox systems of the cells, and thus, the potential role of nanoparticles as antioxidant treatment for several diseases.

**Materials and methods:**

This paper was written after a review of the articles published on the field, using the “PubMed” and “Research Gate” databases.

**Results:**

The main types of nanoparticles are listed and explained below, offering a global vision of the field with great interest for research. Antitumor chemo- and radiotherapies have been found to improve efficacy by enhancing the selectivity of cytocidal effects and minimizing systemic adverse effects when such materials are used. Furthermore, catalytic nanomaterials can execute energy-free antioxidant cycles that scavenge the most harmful reactive oxygen species via SOD- and catalase-like activities.

**Conclusion:**

This unique method is projected to result in significant gains in the long run. However, due to a lack of understanding of potential adverse body reactions to these novel strategies, caution must be exercised. Analyzing the biocompatibility of these nanomaterials carefully, particularly in terms of biokinetics and the problems that could arise from long-term retention of nonbiodegradable inorganic nanomaterials, is required.

## 1. Introduction

The development or etiology of several serious diseases is related to oxidative stress, a situation where the aerobic metabolism produces reactive oxygen species (ROS) inefficiently buffered by antioxidant mechanisms of cells. The overproduction of ROS damages lipids, DNA, as well as proteins, producing cell death and tissue destruction (Beckman and Ames, 1997), and contributing to pathologies such as cancer, chronic inflammation, diabetes, early aging, immuno-deficiencies, ischemia, neurodegenerative diseases, sepsis, viral pathogenesis and a wide range of others (Mashima et al., 2001). Antioxidant therapy has gotten a lot of attention in the last twenty years (Aggarwal et al., 2010), and pharmacological and nutritional strategies to stop or at least mitigate human diseases by boosting antioxidant molecules that increase or imitate endogenous antiradical defenses have been considered very important (Lü et al., 2010). Indeed, the ideal antioxidant therapy remains elusive due to a plethora of intrinsic variables, such as the selection of the appropriate antioxidant system and dosage; excessive ROS scavenging would be deadly because basal ROS are required for proper tissue and cell function (Halliwell and Gutteridge, 2007).

Antioxidant trace elements have been utilized to cure a variety of ailments, for example, hypoxia, a condition that occurs when the body’s oxygen supply is depleted. Nevertheless, in the treatment of human diseases there is a delicate connection between therapeutic and toxic doses of the antioxidant trace element (Lin and Wang, 2005). One technique in order to control the toxicity of the trace element could be the formulation of antioxidant nanoparticles (Tang et al., 2019).

In the last three decades, nanomaterials have received a research interest by the scientific communities because of their unique physicochemical properties (Jeevanandam et al., 2018). Nanoparticles of inorganic origin, consisting of a wide range of transition metals and metal oxides have size- and shape-dependent catalytic properties (Cao et al., 2016). Among all transition metals, manganese oxide has received much attention by the researchers due to the high portion of surface atoms, extraordinary specific surface area and nontoxicity. Their physicochemical properties and wide applications have been studied in bioimaging, biosensing, biosensor, catalysis, drug delivery, energy storage, ion exchange and molecular adsorption (Chen et al., 2019; Ding et al., 2020).

Great opportunities are originated from nanotechnology lines, including redox-active metal oxide nanoparticles, and antioxidant nanoparticles are specially interesting because of their unlimited potential for treatment of human diseases (Celardo et al., 2011). For instance, there is a lot of interest in antioxidant nanoparticles in the treatment of various eye ailments among individuals (Mitra et al., 2016; Apaolaza et al., 2020). In the next pages, we discuss how the state of art is for main antioxidant nanoparticles.

## 2. Role of reactive oxygen species (ROS) in cellular mechanisms

Scientist began to become aware of the existence of a new sort of substance in the later part of the twentieth century because of a study conducted by scientists such as Boveris (Boveris, 1980). It has been shown that they can acquire a variety of positive functions, including cell viability, as scientist learned more about them. The term “Reactive Oxygen Species” was later used to these compounds.

The term “reactive oxygen species” refers to a collection of radicals or molecules that include an oxygen atom. Nitrogenous free radicals, also known as reactive nitrogen species (RNS), and free radicals based on other elements, such as sulfur, are also present. Water is formed when oxygen receives four electrons and is turned into two molecules of water. In biological systems, partial oxygen reduction occurs, resulting in the production of harmful ROS (Yokouchi et al., 2008).

Oxidants have been demonstrated to promote calcium signaling by increasing cytosolic calcium concentrations, implying a putative physiological role for oxidants in Ca^2+^ signaling control (González et al., 2006). In the presence of oxidants, calcium transport through calcium channels increases, and calcium pumps are inhibited, suggesting that these macromolecules could be oxidants’ targets for influencing calcium signaling (González-Flores et al., 2014a).

### 2.1. Role of ROS in apoptosis

The concentration of radical oxygen in the mitochondrial matrix is 5 to 10 times greater than in the cytosol or nucleus (Cadenas and Davies, 2000). The mitochondrial respiratory chain, in addition to being a major source of intracellular ROS, is also a major target for ROS-induced damage; on the other hand, free radicals generated in the mitochondria could inhibit one or more components of the electron transport chain, thereby accelerating the generation of ROS, contributing to the appearance of dysfunction under oxidative stress (González et al., 2010a; González-Flores et al., 2014b; Espino et al., 2020). It is widely known that cellular metabolism is related to a continuous supply of ATP from the mitochondria, essential in most tissues. As a result, any damage to the respiratory chain may have a significant influence on cell viability. The cell has a ROS detoxifying defense mechanism as well as systems to repair the damage that they produce.

Several studies have placed the mitochondria at the focus of apoptosis regulation and its relationship with ROS (Gogvadze et al., 2010). Studies carried out in different cell types indicate that treatment with α-tocopherol succinate (α-TOS), an analog of vitamin E, generates ROS and stimulates a rapid entry of calcium into the cell and consequently into the mitochondria, which was a prerequisite for inducing mitochondrial permeability transition pore (mPTP) (Gogvadze et al., 2010). ROS are capable of indiscriminately oxidizing proteins, lipids, and nucleic acids, altering their structure and function (Ghibelli and Diedrich, 2010). This type of reaction leads to the formation of protein aggregates and facilitates the formation of mPTP, which causes the loss of mitochondrial membrane potential and the release of proapoptotic proteins, such as cytochrome c, from the intermembrane space to the cell cytoplasm, which will initiate the cascade of reactions that culminate in apoptosis or programmed cell death (Ott et al., 2007). Cytochrome c is positively charged and binds to cardiolipin (negatively charged) on the outer leaflet of the inner mitochondrial membrane. The exit of cytochrome c through the mPTP or Bax/Bak pores requires an increase in ROS through a mechanism that may involve the peroxidation of cardiolipin, which causes a change in its physical properties, necessary to break the binding of cytochrome c to cardiolipin, and therefore to the inner mitochondrial membrane (Ott et al., 2002). Oxidative DNA damage causes modifications in the purine and pyrimidine bases, the molecular structure of deoxyribose, single and double chain breaks, as well as creates cross-links in other molecules. Modifications in DNA are potentially mutagenic contributing to the appearance of cancer, neurodegenerative diseases and premature aging. An important ROS target is mitochondrial DNA (mtDNA), which codes for 30 polypeptides and 22 transfer RNAs, which are essential for the electron transport chain and generation of ATP by oxidative phosphorylation. mtDNA is especially susceptible to ROS attack due to its proximity to the electron transport chain and the lack of protective histones. mtDNA contains 10–20 times more oxidatively changed bases than nuclear DNA under oxidative stress. The main source of genomic instability in the mitochondria is oxidative DNA damage, which leads to respiratory failure (González et al., 2010b). Furthermore, one of the most critical aspects in aging is mitochondrial genetic instability.

Direct oxidation and inactivation of iron-sulfur (Fe-S) proteins, such as aconitases, and related iron release are major mechanisms of O_2_^•−^ toxicity. Given the release of iron, it can be conjugated with H_2_O_2_, resulting in the production of hydroxyl radicals that can oxidize proteins, DNA, and mitochondrial lipids, exacerbating the damage caused by O_2_^•−^(Nechushtai et al., 2020).

Mitochondrial aconitase (which possesses an Fe-S center) plays an important role in the Krebs cycle by catalyzing the conversion of citrate to isocitrate. Aconitase inhibition results in cycle dysfunction having a great impact on energy production and cell viability. Complex I NADH dehydrogenase is another Fe-S protein that is affected by the O_2_^•−^. Oxidized proteins are recognized by proteases to be degraded and must be replaced by their de novo synthesis (Ciccarone et al., 2020).

Lipid peroxidation in the mitochondria can cause mitochondrial metabolism to be suppressed. Lipid peroxides have an impact on mitochondrial processes such as oxidative phosphorylation, selective inner membrane barrier characteristics, mitochondrial membrane potential maintenance, and calcium buffering capability (Albano et al., 1991; Bacon et al., 1993; Ayala et al., 2014). Products of mitochondrial lipid peroxidation can interact directly with proteins and/or indirectly with the lipid portion of the inner mitochondrial membrane, affecting its selective barrier characteristic (Chen et al., 1995).

The facilitation of transitory calcium-dependent mitochondrial permeability, which plays a role in certain forms of cell death, is a negative consequence of ROS. In addition to participating in the capacitive entry of calcium (Redondo et al., 2004), ROS generate calcium release from intracellular, mitochondrial, and agonist-sensitive deposits (Pariente et al., 2001). Although mitochondria play an important role in calcium regulation, their capacity is limited due to calcium uptake and retention. If the accumulated calcium exceeds a certain concentration threshold, it will be consequently released from the mitochondria by the mPTP. The calcium threshold of mPTP decreases when calcium reuptake is accompanied by oxidative stress and adenine nucleotide depletion. In fact, it has been proposed that the ROS generated in the mitochondria are directly involved in the induction of mPTP. Thus, both oxidative stress and failure of calcium homeostasis contribute to mitochondrial-mediated cell damage. The mPTP results in mitochondrial failure, which can lead to necrosis due to the depletion of ATP, or also, to a caspase-mediated apoptosis if the mPTP occurs in a subpopulation of mitochondria and others remain intact to produce enough ATP to sustain energy demand required by the apoptosis process (Lemasters et al., 2009).

The relationship between ROS and apoptosis has been shown in various works. Specifically, it has been observed that the treatment of platelets with its physiological agonist thrombin, generates H_2_O_2_ and depolarizes the mitochondrial membrane, consequently inducing apoptosis, events that were inhibited after treatment with catalase.

When platelets were treated with exogenous H_2_O_2_, the release of cytochrome c from the mitochondria and the activation of caspase-9 significantly increased. Because of these events, there was an increase in the activation of caspase-3 and the externalization of phosphatidyl serine (López et al., 2007). Additionally, data have shown that H_2_O_2_ treatment of AR42J rat pancreatoma cells induces an increase in cytosolic [Ca^2+^], mitochondrial depolarization, cytochrome c release, and caspase-3 activation through a mechanism that requires mitochondrial reuptake of calcium (Morgado et al., 2008). In germ cells, such as human sperm exposed to treatment with H_2_O_2_, it has been observed that there is an increase in the activation of caspases −3 and −9 and subsequent externalization of phosphatidyl serine, in a Ca^2+^ -dependent process (Bejarano et al., 2008). These results agree with other in vitro works (Bejarano et al., 2009; Uǧuz et al., 2009; González et al., 2010b; González-Flores et al., 2014b).

### 2.2. ROS and cell growth diseases

It is extensively documented that ROS have a key role in the genesis of two apparently opposite processes, such as apoptosis and cancer. Various oxidants have also been demonstrated to raise cytosolic calcium concentration under subtoxic conditions, implying a possible physiological role for oxidizing agents in the control of calcium signaling (Granados et al., 2006; González et al., 2010a). It should be noted that the different isoforms of SERCA differ in their susceptibility to H_2_O_2_ damage (Redondo et al., 2005).

The participation of ROS in the activation process of T lymphocytes has been described, increasing the immune response and inflammatory processes, and ultimately also causing the activation of signaling pathways that trigger the cell apoptosis process (Castedo et al., 1996). Nitric oxide (NO), for example, has been directly involved in apoptosis, since it produces a decrease in the concentration of cardiolipin and in the activity of the electron transport chain in the mitochondria, which would eventually cause the release of cytochrome c to the cytosol and the induction of apoptosis (Umansky et al., 2000). Other pieces of evidence of ROS activity in the activation of apoptosis are shown in human lymphocytes, where proliferation is inhibited by low doses of O_2_; besides, H_2_O_2_ induces apoptosis, due to the fact that it participates in the formation of the hydroxyl radical (^•^OH). Another example is TNF-α, which has an oxidative effect due to its function and destroys virus-infected tumor cells (González-Flores et al., 2014b). Likewise, H_2_O_2_ has been shown to mobilize calcium from intracellular compartments in different tissues such as rat hippocampal astrocytes (González et al., 2006), mouse pancreatic acinar cells (Pariente et al., 2001) as well as platelets (Jardín et al., 2008), neutrophils (Bejarano et al., 2007), and spermatozoa (Espino et al., 2010).

Mitochondrial respiration generates, in addition to a proton gradient, a substantial amount of superoxide radical, suggesting an interesting correlation among mitochondrial integrity, oxidative stress and the phenomenon of apoptosis (Kim et al., 2004). In fact, there is a Mn^2+^-superoxide dismutase (SOD) in the mitochondria that is responsible for removing the superoxide radicals that originate from the mitochondria, so that when mitochondrial hyperactivity is promoted, due to cellular stimuli that lead to an excessive accumulation of calcium in its interior, the production of radicals is so remarkable that this enzyme cannot buffer it and damage occurs in the mitochondria (Brown et al., 1985).

This superoxide radical created by the mitochondria is turned into H_2_O_2_ both via the enzyme SOD and by spontaneous dismutation. Another significant control of mitochondrial ROS is the mitochondrial membrane potential. ROS generation is clearly correlated with mitochondrial membrane potential (Starkov and Fiskum, 2003), and both chemical uncouplers (for example, 2,4-dinitrophenol) (Okuda et al., 1992) and new uncoupling proteins (UCPs) (Nègre-Salvayre et al., 1997) seem to be able to reduce ROS generation in organs and whole cells, even though in vitro experiments with isolated mitochondria have shown opposite results (Cadenas and Boveris, 1980).

Some authors consider that the power that ROS have to activate apoptotic processes is based on the assumption that they activate the proteins caspase-2 and −9, and inactivate the antiapoptotic factor Bcl-2, through changes in the pH of the mitochondrial matrix (Takahashi et al., 2004).

On the other hand, considering the uncontrolled proliferation of cells that induces the appearance of cancer, ROS also have a direct relationship with this phenomenon, since free radicals induce changes in the DNA sequence, which include phenomena of gene amplification, genetic deletions, point mutations, etc. These alterations activate the expression of various molecules that act as signals in the regulation of metabolism or the regulation of growth, among others.

Under oxidative stress, ROS trigger signaling cascades such as c-Jun amino terminal kinases (JNK), extracellular regulation kinases (ERK), kinases responsible for heat shock (HSPF1), mitogen-activated protein kinase p38 (MAPK), nuclear factor-kappa B (NF-κB) and the phosphatidylinositol 3 kinase pathway (PI3K/Akt) (Johnson et al., 1996; Finkel and Holbrook, 2000); that trigger uncontrolled replication and translation of some genes, and alter the cell cycle causing the cell to divide uncontrollably.

It has also been proven that ROS provide some types of cancer with the ability to mutate, and consequently evade the protective mechanisms of organisms and cause damage to local tissues, such as the invasion of other attached tissues, or even the ability of migrating and producing tumor metastasis (Matés, 1999).

## 3. Antioxidant properties of nanomaterials

**Figure 1:**
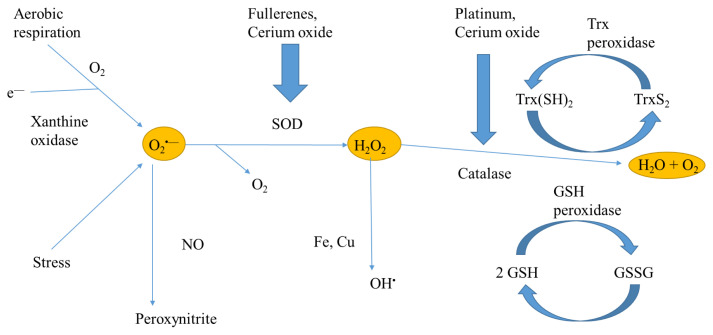
Activity of antioxidant nanoparticles in order to support endogenous antioxidants defenses. GSH: reduced glutathione, GSSG: oxidize glutathione. Trx(SH)_2_: reduced thioredoxin, TrxS_2_: oxidize thioredoxin.

During the last few years, a great research has been carried out in order to develop nanomaterials with outstanding antioxidant efficiency. Figure 1 summarizes the implication of main antioxidant nanomaterials in the oxidative stress signaling pathway. On the other hand, Table 1 shows the main data about the different studied nanomaterials. Further information is included below, in the corresponding subsection.

### 3.1. Nanoceria

The pharmacological effects of cerium oxide nanoparticles (CNPs) remain mainly in the hypothesis, specially sustained by abiotic experiments, which ensured that the nanoscale acts as a regenerative redox system, cycling its state while converting superoxide into hydrogen peroxide, and then the peroxide to water. This antioxidant activity in conjunction with an apparent absence of toxicity makes it an enormously promising therapeutic instrument (Celardo et al., 2011c); yet, both characteristics still have need of clarifications and detailed analyses. Despite of the fact that the regenerative redox cycle has been observed in biological fluids and cells, it should be demonstrated in tissues as well as complete animals. The toxicity must be established in animal models over extended periods of time, especially as the nanoceria cell rescue ability may favor tumor cell persistence, increasing tumor incidence (Celardo et al., 2011b). Nonetheless, results reveal that after a cell is injured, nanoceria enhances nontumor cell survival (four out of five trials), but not cancer cell survival (two out of two studies). Based on these findings, it indicates that using nanoceria as an adjuvant in antitumor therapy could be beneficial, since it could protect healthy cells from antitumor therapies, which are often quite damaging, without compromising tumor cell eradication efficiency (Celardo et al., 2011b).

In the outer layer of CNPs, cerium ions have two valence states (Ce^3+^ and Ce^4+^), which coexist. The creation of oxygen vacancies, which offer inherent antioxidant properties to CNPs, can compensate for the charge shortfall given to Ce^3+^ (Celardo et al., 2011a, c; Das et al., 2013). The 3+/4+ valence switch in nanoceria is similar to the function of redox enzymes, which use metals as cofactors to trigger reversible redox reactions in tissues and cells. CNPs work in a similar way to naturally occurring metalloenzymes, which scavenge ROS in cells and tissues using transition metal cofactors as Cu, Fe, Mn, or Zn.

Because only Ce^3+^ may be oxidized and produce peroxide, superoxide reduction requires nanoceria with a high Ce^3+^/Ce^4+^ ratio. This process is named SOD-mimetic because it mimics the reduction of superoxide by SOD. Reestablishing the reduced state of the metal present in SOD necessitates enzymatic recycling; it has been proposed that nanoceria may “spontaneously” recycle, via a still unclear mechanism (Das et al., 2007). Considering the catalase-(Pirmohamed et al., 2010) and the SOD-mimetic (Heckert et al., 2008) activities together, we may imagine a bio-related mechanism of restoration of nanoceria (Celardo et al., 2011c). When superoxide is reduced, H_2_O_2_ is produced, and Ce^3+^ is oxidized to Ce^4+^. The reaction between Ce^4+^ and H_2_O_2_ can then recycle Ce^3+^ and oxidize H_2_O_2_ to O_2_ (Celardo et al., 2011b). This mechanism may regenerate reduced nanoceria and eliminate, in a sequential set of chemical reactions, both superoxide and hydrogen peroxide (Celardo et al., 2011c). For each H_2_O_2_ oxidized, the stoichiometry requires the reduction of two superoxides, (Das et al., 2007; Heckert et al., 2008) resulting in the formation of Ce^4+^ and the reduction of H_2_O_2_ to H_2_O; in this case, a real catalase-like dismutation cycle is formed.

CNPs have been shown to resemble superoxide dismutase when the Ce^3+^ form combines with superoxide, converting it to Ce^4+^ while also converting superoxide to hydrogen peroxide (Heckert et al., 2008). Similarly, oxidation of Ce^3+^ to Ce^4+^ ions permits scavenging other toxic reactive species such as hydroxyl radicals (Xue et al., 2011), NO (Dowding et al., 2012) and peroxynitrite (ONOO-) (Dowding et al., 2013). Alternatively, Ce^4+^ can be converted to Ce^3+^ by oxidizing hydrogen peroxide to molecular oxygen, like how the catalase enzyme reduces hydrogen peroxide to molecular oxygen (Pirmohamed et al., 2010). As a result, the Ce^3+^/Ce^4+^ pair can reversibly switch back and forth while scavenging superoxide and peroxides (Celardo et al., 2011a, c; Das et al., 2013). It has been suggested that CNPs suffer a whole redox cycle while scavenging two superoxides and one hydrogen peroxide. This nanomaterial’s self-regenerating antioxidant activity is a potentially irreplaceable pharmaceutical prospect, making it a unique and intriguing ROS biological scavenger (Caputo et al., 2014).

**Figure 2:**
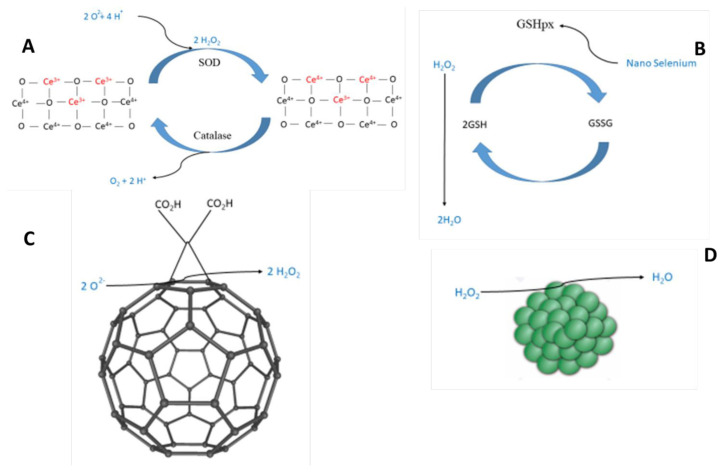
Antioxidant mechanisms of several nanomaterials. A) Cerium oxide particles (CNPs) redox regenerative system using SOD-and catalase-like reactions. B) Selenium particles (SeNPs) boosting glutathione peroxidase activity. GSHpx: glutathione peroxidase; GSH: reduced glutathione, GSSG: oxidize glutathione. C) Mechanism of action of fullerene. Apart from direct scavenging of free radicals, this nanomaterial shows SOD-like activity. D) Platinum particles (PtNPs) activity scavenging hydrogen peroxide. This nanomaterial shows an interesting catalase-like activity.

The Ce^3+^/Ce^4+^ ratio in CNPs varies depending on the synthesis procedures: for example, hexamethylenetetramine or base (sodium hydroxide or ammonium hydroxide) coprecipitation techniques yield 21%–30% surface concentrations of Ce^3+^ ions, whereas hydrogen peroxide as a synthesis cofactor yields higher concentrations (55%–65%) (Das et al., 2013). For that reason, CNPs capacity to preferentially react with different free radicals may be controlled changing the method of synthesis and the Ce^3+/^Ce^4+^ ratio. Although CNPs with higher quantities of Ce^3+^ ions have a stronger SOD-like action, there is substantial evidence that CNPs with greater Ce^4+^ (70%–80%) concentration have a stronger catalase mimetic effect (Pirmohamed et al., 2010; Celardo et al., 2011c). As a result, if the Ce^3+^ surface concentration is lowered to 5%, the SOD-like effect is completely lost (Heckert et al., 2008). Consequently, an exact balance between the two valence states is required for catalytic vs. stoichiometric redox activity, because the self-regenerating antioxidant cycle requires both catalase and SOD-like functions to be active at the same time (Caputo et al., 2014). Figure 2A describes briefly the mechanism of action of CNPs.

In recent studies, spectroscopic studies of Ce^3+^/Ce^4+^ ratio on several stages of nanoceria-oxidant interaction have demonstrated the crucial contribution of oxygen transport in the redox processes for ceria nanoparticles (Malyukin et al., 2018). Oxygen diffusion is a sufficient limiting factor in the processes of self-regeneration of antioxidant properties of ceria nanoparticles following oxidation, according to an analysis of nanoceria antioxidant activity during OH scavenging. Both size reduction and temperature rise improve the rate of oxygen transport, allowing nanoceria antioxidant capabilities to regenerate more quickly (Malyukin et al., 2018).

Moreover, the pharmacological potential of nanoceria has been evaluated demonstrating their worth in a biological system. As shown in Table 2, the pretreatment with nanoceria in human histiocytic lymphoma U937 cells was not lethal, and decreased the generation of intracellular ROS, mitochondrial depolarization and the percentage of apoptotic cells in a more potent way than any of the classical antioxidants tested, such as N-acetyl cysteine (NAC) or trolox (González-Flores, 2014a). In this study, the percentage of apoptosis was reduced by nanoceria, indicating that there is an effect of intracellular ROS generation in TNFα-induced apoptosis in combination with cycloheximide (CHX). Moreover, the flow cytometry studies revealed that the intracellular ROS generation was amplified because of the administration of TNFα + CHX. Nevertheless, preincubations of cells with nanoceria were able to revert significantly the ROS generation and mitochondrial depolarization induced by the cotreatment of cells with TNFα + CHX.

These effects agree with previous report (Celardo et al., 2011a), which used etoposide (VP-16), hydrogen peroxide, and puromycin to induce apoptosis in the same cell line U937. Similarly, nanoceria was found to scavenge free radicals in cultured retinal neurons treated with H_2_O_2_ (Chen et al., 2006), in murine insulinoma cells treated with hydroquinone (Tsai et al., 2007), and normal human colon cells (Colon et al., 2010). Every day, new nanoceria uses in the emerging field of nanomedicine arise, such as selective medication targeting and tissue engineering (Celardo et al., 2011a).

Nanoceria have synergistic toxic effects in cancer cells when functionalized with anticancer drugs (Xu and Qu, 2014). On human dermal fibroblasts, nanoceria reduced the toxicity of the anticancer medication doxorubicin (Zschauer et al., 2017). Nanoceria has been shown to have protective effects on healthy cells while killing glioma cancer cells (Minotti et al., 2004). Furthermore, nanoceria was found to successfully scavenge the excessive ROS generated in the arthritic joint. The immunomodulatory and antiapoptotic roles of nanoceria were checked in parallel with in vitro experiments, principally involving osteoarthritis-mimicking chondrocytes/macrophages coculture models, where nanoceria immunomodulatory and antiapoptotic roles were revealed to be main molecular mechanisms. The current findings suggest that nanoceria could be a hopeful nanotherapeutic candidate for treating degenerative joint disorders (Dashnyam et al., 2021).

On the other hand, nanoceria multi-enzyme-like capabilities have been effectively applied to biological detection and analysis, such as colorimetric immunoassays, enzyme-linked immunosorbent assays (ELISA), and biosensors, among other things (Xu and Qu, 2014; Song et al., 2018; Liao et al., 2019; Ni et al., 2019).

### 3.2. Selenium

Among the antioxidant nanoparticles, selenium (Se) nanoparticles (SeNP) appeared as capable tools in order to combat the hypoxia-induced in several diseases (Soumya et al., 2014; Amani et al., 2019). Se is a micronutrient and essential major trace element for mammalian metabolism, due to its exceptional pharmacologic and physiologic functions for decreasing eye diseases (Higuchi et al., 2012; Pellegrini et al., 2020). Indeed, Se controlled the intensifications of apoptosis, Ca^2+^ influx and mitochondrial oxidative stress, in several cells (Uǧuz et al., 2009; Soumya et al., 2014; González De Vega et al., 2018). Additionally, Se reduced ischemia/reperfusion-induced oxidative injury in retina of rats (Yazici et al., 2015; Duzgun Ergun et al., 2020). The retina is very vulnerable to oxidative damage when it is exposed to too much light, whether it is hypoxic (Masuda et al., 2017).

Selenoenzymes, the glutathione peroxidase family (GPXs), and thioredoxin reductase (TR) are all involved in the antioxidant impact of selenium nanoparticles (Kondaparthi et al., 2019). In comparison to selenocysteine, selenite, selenomethionine and Se-methyl, selenium nanoparticles can boost selenoenzyme activity with equivalent efficacy and lower toxicity (Khurana et al., 2019). Figure 2B represents the mechanism of action of SeNP.

Accumulating data point out that SeNPs have key defensive roles in hypoxia-induced neuronal injury (Ali et al., 2020). Recent research has shown that the PARP-1 enzyme has a role in inflammation and hypoxia-induced oxidative cytotoxicity in ARPE-19 cells (Kovacs et al., 2019). In HEK293 cells, a modulatory role of Se on hypoxia-induced increase of cytosolic [Ca^2+^]^c^ via suppression of TRPM2 was recently revealed (Kovacs et al., 2019). By potentiating TRPM2 in DBTRG glioblastoma cells, Se was found to have a positive effect on chemotherapeutic agent-induced elevations in cytosolic [Ca^2+^]_c_ and PARP-1 activation (Ertilav et al., 2019). SeNP was also discovered to have a modulatory effect in the hypoxia-induced activation of TRPM2 and PARP-1 in ARPE-19 cells a few months ago (Özkaya et al., 2021). Therefore, the effects on PARP-1 and TRPM2 have been established in different cell lines, such as ARPE-19, DBTRG, HEK293 and SH-SY5Y cells (Ertilav et al., 2019; Kovacs et al., 2019; Akyuva and Naziroğlu, 2020; Duzgun Ergun et al., 2020).

Mitochondria have a key role in the production of ROS. Accumulating proofs show that the increase of Ca^2+^ into mitochondria leads to a disproportionate production of ROS via the increase of mitochondria depolarization (Uǧuz et al., 2009; Carrasco et al., 2015). In earlier studies, excessive ROS generation via upregulation of mitochondrial ROS, mitochondrial depolarization and TRPM2 activation markers in the hippocampus and microglia were induced by hypoxia (Ataizi et al., 2019; Akyuva and Naziroğlu, 2020). Though, these anomalous processes were inhibited by the treatment of Se (Ataizi et al., 2019; Akyuva and Naziroğlu, 2020). Recent data indicate that hypoxia stimulated mitochondrial depolarization and mitochondrial ROS processes. Nonetheless, SeNP administration reduced ROS and mitochondrial depolarization processes in ARPE-19 cells via inhibiting TRPM2 (Özkaya et al., 2021). It gives the impression that the intensification of mitochondrial depolarization via the increase of TRPM2 dependent Ca^2+^ influx into mitochondria leads to extreme ROS generation. This pathway was boosted by hypoxia, which increased ROS production and accelerated TRPM2 activation, mitochondrial ROS, and mitochondrial depolarization, leading to more ROS production and an ARPE-19 cell death cycle. The TRPM2 modulatory feature of SeNP is thought to be able to suppress these atypical pathways (Özkaya et al., 2021).

### 3.3. Carbon

Developments in nanotechnology have provided new materials with several possibilities. Carbon nanomaterials (CNMs) are a type of material that has been employed in a variety of applications, including agriculture (Verma et al., 2019). These CNMs have the property of being easily absorbed by plant cells, resulting in favorable impacts on plant development and growth (Ghorbanpour and Hadian, 2015). Among the application highlights are the capacity to eradicate heavy metals from water and soil (Fiyadh et al., 2019), antifungal and bactericidal effects and high potential for the removal of pesticides in water (Dehghani et al., 2019). In agriculture, they have been used to induce seed germination (Verma et al., 2019), and they also enhance the antioxidant activity of plants (Ghorbanpour and Hadian, 2015), and act as growth regulators (Patel et al., 2018). Among the most studied CNMs in agriculture, carbon nanotubes, fullerene, graphite and graphene are found (Fiyadh et al., 2019). It has been shown that both carbon nanotubes and graphene are biocompatible materials (Liu et al., 2013; Andelkovic et al., 2018), and consequently, they can be applied in plants.

The use of carbon nanoparticles in both drench and foliar applications changes the antioxidant defense system of tomato seedlings in a good way. Nevertheless, for each specific antioxidant compound, the outcomes were different depending on the doses used, the route of application (drench or foliar), as well as the type of carbon nanomaterial used (graphene or carbon nanotubes) (González-García et al., 2019). However, it has also shown toxicity after exposure to different carbon nanomaterials (Raja et al., 2019).

On the other hand, buckminsterfullerene (frequently called fullerene) is a nanostructure that in 1985 was firstly identified; it is an innovative allotropic pure crystal form of carbon like graphite and diamond that has a fixed number of atoms, which is very uncommon among nanoparticles, and as a result it can be classified as a molecule (Caputo et al., 2014). Fullerene and their water-soluble derivatives scavenge a lot of ROS including hydrogen peroxide (Yin et al., 2009), hydroxyl radical (Djordjevic et al., 2005), nitric oxide (Mirkov et al., 2004), singlet oxygen (Yin et al., 2009), and superoxide (Yin et al., 2009). Direct scavenging of free radicals R, which involves their direct reactivity with fullerenes C55C doubly bonded, resulting in the production of a C_60_-RH complex (Djordjevic et al., 2005); or a catalytic reaction (e.g., catalase- and SOD-like activity) that does not involve a chemical change of the fullerene surface (Ali et al., 2004). In Figure 2C it can be observed the mechanism of action of fullerene. The SOD-like catalytic activity of fullerenes protects brain cells derived from genetically defective SOD2 mice from oxidative damage by providing biologically operative mitochondrial SOD activity, thus acting as SOD mimics (Ali et al., 2004). Carboxyfullerene’s SOD mimetic activity has been shown to improve cognition and lengthened the longevity of transgenic mice when consistently supplied in their drinking water (Quick et al., 2008). The antioxidant defense of fullerene derivatives was shown in mice and cell culture challenged with different kinds of oxidative stress-inducing products such as deoxy-D-ribose (Fumelli et al., 2000), glutamate (Jin et al., 2000), hydrogen peroxide (Tsai et al., 1997), ionizing radiation (Daroczi et al., 2006) and ultraviolet light (Fumelli et al., 2000).

Fullerene indicates pharmacological activity in the treatment of several other oxidative stress related diseases such as macular degeneration, ischemia, excitotoxicity and stroke as confirmed in cell culture (Dugan et al., 1997, 2001; Lotharius et al., 1999; Bisaglia et al., 2000; Tzeng et al., 2002) and in vivo (Lin et al., 1999, 2002). Specially, hexasulfobutylated fullerenes exerted substantial cardioprotection in coronary occlusion/reperfusion injuries via their free radical scavenging action (Lin et al., 2002). In vivo investigations have shown that fullerenol can scavenge the free radicals that are produced in the small intestine after ischemia/reperfusion injury in dogs (Younglai et al., 2001). In mice with ischemia with reperfusion lung models, fullerenol C(60)(OH)(24) derivatives protect them from oxidative damage (Chen et al., 2004).

Because of their unique physicochemical features, nanodiamonds (NDs) have several advantages in nanotechnology and they must be considered in this review. NDs are useful in a variety of sectors, including nanomedicine, nanocosmetics, and biomedicine, due to their chemical stability, natural fluorescence, and high absorption capacity. According to recent research, NDs can scavenge ROS and hence act as antioxidants. However, research on the antioxidant activities of NDs is limited, and their impact on oxidative stress caused by hydrogen peroxide (H_2_O_2_) is unclear. Furthermore, toxicity studies are required due to the increased use of cNDs in nanomedical, nanocosmetics, and biomedical goods. In (Kaluç and Thomas, 2021) the effects of a carboxylated ND (cND) on H_2_O_2_-induced oxidative stress and its 24-h toxicity in Saccharomyces cerevisiae, a unicellular eukaryotic model, were investigated. By lowering H_2_O_2_ levels, cND dramatically lowers cell mortality and ROS generation in response to H_2_O_2_-induced oxidative stress. Even though higher concentrations inhibited colony formation, even 10.000 g/mL cND treatment for 24 h could not entirely prevent colony formation. cND also lowered the amount of ROS produced during normal metabolism without causing cell death. Thus, it can be concluded that cND has ROS-scavenging function but no toxicity (Kaluç and Thomas, 2021).

Finally, another form of carbon, carbon dots (CDs), seems to have an interesting future. The ability of carbon dots to scavenge or generate ROS has been controlled by integrating heteroatoms (Cu and Cl ions) (Getachew et al., 2021). Cu and Cl codoped CDs (CuCl-CDs) possessed not only ROS generation ability upon laser irradiation for photodynamic therapy (PDT), but also peroxidase-mimic activity that generates oxidative •OH from hydrogen peroxide (H_2_O_2_) for chemodynamic therapy, with half-maximal inhibitory concentrations (IC_50_) of •O_2_ and •OH radicals estimated to be 6.89 and 6.12 g/m (CDT). Furthermore, the colorimetric assay, 1O_2_ emission peak, and ESR data all indicated that •O_2_, •OH, and 1O_2_ radicals were produced efficiently. CuCl-CDs with ROS-generating and peroxidase-mimetic characteristics were also successfully combined with polydopamine (PDA) and glucose oxidase (GOx) to form multifunctional GOx/CuCl-CD@PDA-PEG (GCP) nanocomposites. The combination of H_2_O_2_ and laser irradiation yielded a substantial output of ROS and these novel GCP nanocomposites had good photothermal conversion efficacies. Furthermore, the presence of GOx in GCP nanocomposites allows these chemicals to lower intracellular glucose levels for starving therapy while also increasing enzymatic cascade activity for improved ROS-mediated therapy. In vitro experiments indicated that these GCP nanocomposites were biocompatible at concentrations ranging from 100 to 1000 ppm, but not at 200 ppm (Getachew et al., 2021).

### 3.4. Platinum

When we arrive to the nanoscale, the typically inert noble metals (e.g., palladium, platinum, gold) begin to show remarkable catalytic properties (Narayanan and El-Sayed, 2004; Rothenberg, 2010; Zhou et al., 2010). In comparison to silver, gold, and other metal NPs, PtNPs are one of the most studied nanoparticles (particularly for catalysis), and the diversity of synthesis approaches is very similar to AuNPs (Jeyaraj et al., 2019). Bacteria, fungi, and plants are examples of biological systems that could be used as environmentally friendly nanofactories (Bloch et al., 2021). Green synthesis is a viable alternative to physical and chemical synthesis because it is nontoxic, cost-effective, provides rapid synthesis, is environmentally friendly, monodispersed, produces minimal waste, and allows for large-scale production (Jeyaraj et al., 2019). In fact, several bacteria have the skill to biosorb metal ions on their surfaces and ultimately reduce them to nanoparticles through a variety of methods that include reductases, cytochromes, and metallothioneins (Puja and Kumar, 2019; Kumar et al., 2020). Anaerobic sulphate reducing bacteria, rhizospheric bacteria, bacterial cellulose, and even photoautotrophic cyanobacteria can all produce PtNPs with unusual shapes and sizes. The bacterial cell enzymatic and nonenzymatic methods for reducing Pt ions to PtNPs provide better control over shape, size distribution, and crystallinity (Bloch et al., 2021).

In biomedical applications, bacteriogenic PtNPs have a bright future, particularly in the areas of drug administration, gene delivery, early illness diagnostics, cellular and deep tissue imaging, targeted therapy, and multifunctional therapies (Bloch et al., 2021). Above all, PtNPs exhibit strong antioxidant properties, making them a promising material for the pharmacological treatment of oxidative stress-related illnesses and tumors (Espino et al., 2020; Gutiérrez-Tarriño et al., 2021). Many different ROS, such as hydrogen peroxide, hydroxyl, and superoxide, have been demonstrated to react with PtNPs in vitro (Kajita et al., 2007; Hamasaki et al., 2008).

PtNPs made from the supernatant of different bacteria microbial cell lysate demonstrated antioxidant and antibacterial properties. The antioxidant potential was assessed using 2,2-diphenyl-1-picrylhydrazyl (DPPH) radical scavenging activity, whereas the antibacterial property was assessed using the minimum inhibitory concentration (MIC). In the presence of PtNPs, the purple color of DPPH was transformed to pale yellow, showing that PtNPs participates in electron/hydrogen transfer and neutralizes the DPPH radical. It was discovered that antioxidant activity is dosage dependent. Various microbial strains demonstrated varying antioxidant activity when exposed to PtNPs at a concentration of 1000 g/mL (Bloch et al., 2021). The strain ZCI5 has the highest antioxidant activity, followed by FZC6, CCV1, NRRL B-11177, KC19, KT2440, ADR19, and MN23. FZC6 and ZC15 strains had 95% DPPH free radical scavenging activity, while KC19, NRRL B-11177, KT2440, and CCV1 strains had 70% activity (Bloch et al., 2021).

PtNPs, like CNPs, have SOD and catalase activity (Kajita et al., 2007; Yoshihisa et al., 2011), giving them intriguing biological features. However, the process at the basis of catalase and SOD-like catalytic activity of nanoplatinum must be examined, as far as we know. Figure 2D shows the promising catalase-like activity.

### 3.5. Manganese oxide

The biological consequences of nanoparticles of manganese oxide were studied by (Zaitseva et al., 2013) after 30 days of intragastric treatment in Wistar rats. Body mass loss, activation of oxidation processes (increased levels of lipid hydroperoxides, MDA in the blood serum), decrease in antioxidant activity (inhibited antioxidant activity in the blood serum), damaged hepatocyte membranes (higher serum AST and ALT levels), and protein synthesizing liver function abnormalities were among the side effects (low albumins, high gamma globulins in the blood serum) (Zaitseva et al., 2013).

However, very recent results using DPPH and nitric oxide (NO) assay have demonstrated that the scavenging action of manganese oxide nanoparticles is dose-dependent in MCF-7 cell line (Tabassum et al., 2021). 1,1-diphenyl-2-picryl hydroxyl (DPPH) is an easy and fast way to assess antioxidant properties using a spectrophotometer. A hydrogen atom from the antioxidants is accepted by the odd electron in DPPH, which turns it into identical hydrazine. The scavenging activity of nitric oxide was measured using the capacity for inhibition in the generation of nitrite with oxides and oxygen. The results showed that manganese oxide nanoparticles have more DPPH and nitric oxide scavenging activity at 800 μg/mL concentration (Tabassum et al., 2021). Previous results have also shown the radical scavenging ability of biosynthesized MnO2 nanoparticles (Sivanesan et al., 2017). These results highlight the potential of manganese oxide nanoparticles. Therefore, it can be postulated that the mixed phase of manganese oxide nanoparticles are an effective candidate for biomedical applications. However, more research must be done in order to elucidate the mechanism and the real potential of these nanoparticles.

### 3.6. Palladium

The coordination chemistry of Pd(II) and Pt(II) complexes is strikingly similar, prompting research into Pd(II) molecules as anticancer medicines (Coskun et al., 2013; Kapdi and Fairlamb, 2014). Pd(II) complexes have a ligand exchange rate that is roughly 104–105 times faster than Pt(II) counterparts, although they have superior solubility than Pt(II) complexes (Coskun et al., 2013). Previous research has found that ligand selection is critical, since they play a vital influence in a variety of concerns such as reactivity, lipophilicity, and stability (Bugarčić et al., 2015). Various Pd(II) compounds with promising anticancer action have been identified in this regard (Kapdi and Fairlamb, 2014; Ćoćić et al., 2017; Qin et al., 2018; Gutiérrez-Tarriño et al., 2019; Espino et al., 2020).

Chemical and thermal stability, electrical characteristics, and optical properties of palladium nanoparticles (PdNPs) are all significant (Chen and Ostrom, 2015; Saldan et al., 2015). They could also be biofunctionalized in order to be used in medicine (Bharathiraja et al., 2018; Zhou et al., 2018). In addition, photothermal agents, drug transporters, and prodrug activators have all been employed with PdNPs. Antimicrobial, antioxidant, and cytotoxic properties have been discovered in them (Saldan et al., 2015; Liu et al., 2016; Azizi et al., 2017). Several papers described the synthesis of PdNPs employing hazardous, expensive, and multistep traditional procedures such chemical reduction, sol-gel as well as electrochemical and chemical precipitation (Chen and Ostrom, 2015; Saldan et al., 2015).

However, the biosynthesis of PdNPs has been recently carried out using microorganisms, algae and plant extract in an eco-friendly, clean, and safe approach to produce NPs of versatile shapes, sizes, chemical, biological and physical properties (Siddiqi and Husen, 2016; Phan et al., 2019).

PdNPs mediated by biological systems demonstrated bactericidal action against Gram-negative and Gram-positive bacteria, antioxidant, antifungal, and anticancer activity against cancer cells coming from cervical, breast, ovarian, and lung malignancies, among other biomedical uses. In comparison to platinum-based anticancer medicines like carboplatin, cisplatin and oxaliplatin, PdNPs capped with plant extracts are nontoxic and biocompatible. Furthermore, when compared to other synthetic anticancer medicines, biosynthesized PdNPs were revealed to have higher increased anticancer properties. These discoveries could lead to the creation of new antibacterial and anticancer medications that are both effective and safe (Fahmy et al., 2020).

## 4. Conclusions

Nanomedicine is a growing field where novel applications are appearing, especially drug targeting and tissue engineering (Celardo et a., 2011). The advantages of handling particles instead of molecules improve significantly the control of the biochemical outcomes (Caputo et al., 2014). It is also important the ability of some of these nanomaterials to discriminate between normal and tumor cell, which increases the potential and interest of this field.

According to the studied works, nanoceria seems to be a very useful nanoparticle, in terms of antioxidant activity, due to the SOD- and catalase-like activity. Depending on the dose, duration, frequency, and oxidation state of selenium nanoparticles, they have different antioxidant and pro-oxidant effects. The selenium nanoparticle safety profile is not yet well defined, which limits their application. There is an extensive literature on the biomedical applications of carbon nanomaterials. However, plant models are an important focus for carbon derivatives, but more research about the toxicity in plants must be done. It is important to highlight that there are other nanomaterials which are currently being developed, so it might be possible that the list of nanomaterials with antioxidant properties grows in a short period of time.

The next step for the most promising nanomaterials could be a deep analysis of biocompatibility. A strong documentation about the long-term impact of these nanomaterials is necessary, but it is not still produced. Although there are important experimental difficulties, this step is compulsory in order to get a risk/benefits balance and thus go further with this promising field.

## Figures and Tables

**Table 1:** Summary of antioxidant materials considered in this review.

Nanoparticle	Models	References
Cerium oxide	BEAS-2B, J774A.1, A549, RAW 264.7, HCT 116, U937 and Jurkat cells	[18, 59, 63–67, 71, 72, 76–79]
Selenium	Cancer (cervical, uterine), A549, ARPE-19, HepG-2 and MCF-7 cells	[6, 7, 41, 80, 81, 83–86, 88–93, 96]
Carbon nanotubes	Tomato seedlings, *Satureja khuzestanica*,	[98–101, 105]
Fullerene	Keratinocytes, mice, RAW 264.7 cells	[96, 106, 109–111, 114, 115, 118, 120, 121]
Graphene	Tomato seedlings	[102, 103]
Platinum	U937 and HH cells	[128–135]
Manganese oxide	MCF-7 cells	[10, 136–138]
Palladium	U937 and HL-60 cells	[22, 139, 151, 152, 140, 142–147, 149]

**Table 2:** Effect of nanoceria on TNF-mediated apoptosis in combination with cycloheximide (CHX) in U937 cells. Mitochondrial depolarization (ΔΨ_m_), reactive oxygen species (ROS) generation and % apoptotic cells were determined. ROS generation was measured using two different fluorimetric dyes: 2′,7′-dichlorofluorescin diacetate (DCF) and dihydrorhodamine 123 (DHR). Cells were also preincubated with three different antioxidants: Trolox, N-acetyl cysteine (NAC) and nanoceria. ↑ represents an increase and ↓ represents a decrease (González-Flores et al., 2014).

Treatment	Results			
	ΔΨ_m_		ROS (DCF)	ROS (DHR)
TNF+CHX	↓		↑	↑
TNF+CHX+Trolox	↑		↓	↓
TNF+CHX+Nanoceria	↑		↓	↓
	**% Apoptotic cells**			
	**1 h**	**2h**	**3h**	**4h**
TNF+CHX	11%	35%	40%	55%
TNF+CHX+Trolox	11%	20%	30%	32%
TNF+CHX+NAC	15%	25%	35%	40%
TNF+CHX+Nanoceria	11%	10%	22%	21%
